# Space–time interference in action

**DOI:** 10.1186/s41235-026-00745-7

**Published:** 2026-07-21

**Authors:** Cindy Jagorska, Milena Michel, Martin Riemer

**Affiliations:** 1 Biological Psychology and Neuroergonomics, Technical University Berlin, Fasanenstr. 1, 10623 Berlin, Germany; 2 Bernstein Center for Computational Neuroscience (BCCN), Berlin, Germany; 3 Center for Behavioral Brain Sciences (CBBS), Magdeburg, Germany

**Keywords:** Space–time interference, Perception versus action, Motor behavior, Virtual reality

## Abstract

Space–time interference has been frequently reported in perceptual paradigms, but it is unclear to what extent these effects generalize to action-related tasks. We therefore investigated whether space–time interference extends to the execution of goal-directed reaching movements towards 3D objects presented in immersive virtual reality. Forty participants performed both perceptual and motor judgments with respect to the spatial and temporal occurrence of sequentially from left to right presented 3D spheres, with spatial and temporal interstimulus intervals systematically varied. Consistent with previous findings in 2D paradigms, we observed bidirectional interference in a goal-directed pointing task: larger spatial intervals delayed temporal predictions (positive space-on-time effect), whereas longer temporal intervals shifted spatial predictions leftward (negative time-on-space effect). Perceptual judgments showed a significant positive time-on-space effect, but no space-on-time effect. Interference effects in perceptual and pointing tasks were not correlated. Our findings demonstrate that space–time interference extends to more complex motor behavior, highlighting its relevance for everyday actions such as reaching, catching, and navigating.

## Significance Statement

Integrating spatial and temporal information is vital for everyday actions such as catching a ball or crossing a street, and cross-dimensional interference between space and time might influence these actions. Despite frequent real-world examples, most research on space-time interference has been limited to perceptual tasks, even though it is known that perception and action rely on partly distinct neuronal mechanisms. Consequently, it remains unclear whether space-time interference also affects motor behavior. The present study overcomes these limitations by using immersive virtual reality to examine space-time interference during ballistic reaching movements. This approach provides a more naturalistic test of how spatial and temporal information interact during real-world action. As perceptual space-time interference has been observed across the lifespan and may increase with age, our findings establish an important basis for exploring how space-time interference influences motor performance in everyday life.

## Introduction

It has been demonstrated that judgments regarding time and space can be influenced by properties of the respective other dimension. For example, time is judged to last longer in larger rooms while the room size is judged to be larger when more time has been spent in a room (Bogon et al., 2025; Jagorska et al., 2025). A similar interference between time and space has been demonstrated in the field of spatial navigation, with judgments on travel time and traveled distance influencing each other (Cohen et al., 1963; Gladhill et al., 2022, 2024; Jagorska & Riemer, 2024). Interference between time and space has also been found for more abstract stimuli, such as 2D geometric forms displayed on a desktop screen (Cai et al., 2018; Coull et al., 2015; Martin et al., 2017; Xuan et al., 2007). Researchers often refer to everyday examples such as catching a ball or crossing a busy street to highlight the importance of spatio-temporal integration mechanisms (Coull et al., 2013; Desme et al., 2024; Grondin, 2010; Kruijne et al., 2022; Maaß et al., 2022; Mioni et al., 2024). In light of these action-related examples, it is surprising that most studies on space–time interference focus on mere perceptual judgments, rather than goal-directed actions. Catching a ball or crossing a busy street requires coordinated motor behavior going beyond purely visual processing. This discrepancy is unfortunate, because it has frequently been shown that biases in perceptual judgments do not always coincide with an altered motor performance (Schenk & McIntosh, 2010). For example, Aglioti et al. (1995) demonstrated in an experiment that the Ebbinghaus illusion (i.e., a circle is perceived as smaller in diameter when surrounded by relatively large distracter circles) did not influence the pinching width, when the participants were instructed to grasp a circular object. Therefore, the question emerges whether space–time interference is also observable during active motor behavior.

Studies on space–time interference in action-related contexts are sparse. One example is Schroeger et al. (2022), who investigated space–time interference in an interception task. Participants were presented with three consecutively appearing dots, presented from left to right on a 2D computer display, with both spatial and temporal interstimulus intervals held constant within a given trial. Participants were instructed to intercept an imagined fourth dot by indicating both *when* and *where* it would appear via a timed mouse click at the expected screen location. Across trials, the spatial and temporal interstimulus intervals were systematically varied between 150 and 350 pixels, and between 700 and 1500 ms. The results revealed that when the spatial interstimulus interval was larger, participants tended to intercept the imagined fourth dot at a later point in time, relative to trials with smaller spatial intervals, demonstrating a space-on-time effect in an action-related context. Schroeger et al. (2022) also found that increased temporal interstimulus intervals were associated with participants intercepting the imagined fourth dot further to the left (i.e., against the direction of motion), demonstrating a negative time-on-space effect. This negative time-on-space effect resonates with the representational momentum effect, the well-studied tendency to represent the final position of a moving object as slightly displaced in the direction of motion (Freyd & Finke, 1984; Hubbard, 2005, 2015). As the representational momentum effect is attenuated at lower implied speeds (Merz et al., 2023), increased temporal interstimulus intervals (and hence reduced implied speed; Reali et al., 2019) should diminish a predictive spatial shift in the direction of motion, reinforcing a relative leftward bias and hence negative time-on-space effect.

While the study by Schroeger et al. (2022) demonstrates that space–time interference can manifest in motor behavior, it was conducted using 2D stimuli, which limits the generalizability of their findings to real-world situations involving 3D objects. Research on motor coordination during grasping movements has shown that perceptual biases are more prone to transfer to motor behavior when interacting with 2D stimuli compared to 3D stimuli. For example, it was found that the manipulation of irrelevant object features decrease the precision of grasping width for 2D but not for 3D objects (Freud & Ganel, 2015; Ganel et al., 2020). Therefore, in spite of many anecdotal notions, it is currently unclear whether space–time interference also manifests in the behavioral interaction with 3D stimuli.

To investigate space–time interference in active motor behavior within a three-dimensional environment, we adapted the paradigm used in Schroeger et al. (2022) by using immersive virtual reality techniques. As in Schroeger et al. (2022), participants were presented with three spheres from left to right along an imaginary horizontal line and judged the temporal onset or spatial location of a fourth sphere. Participants were asked for perceptual judgments as well as for active, self-paced reaching movements towards 3D objects presented within a virtual environment. If space–time interference manifests in the interaction with 3D stimuli, we would expect similar results as in Schroeger et al. (2022). Specifically, an increase of the spatial interstimulus interval should bias participants’ temporal predictions to a later onset, whereas increasing the temporal interstimulus interval should negatively bias participants’ spatial predictions towards a more leftward location.

## Methods

### Participants

A total of 41 participants were recruited via the SONA recruitment system of the Technical University of Berlin. The data from one participant had to be excluded due to technical issues with the data recording. The final data set consisted of 40 participants (20 female, 20 male; mean age = 26.83 years, SD = 3.25). All participants had normal or corrected-to-normal vision. All participants gave written informed consent and were compensated either financially or with course credits. The experimental procedure was approved by the local ethics committee of the Technical University Berlin (certificate number: 2358322). The required sample size was approximated using G*Power (Version 3.1.9.4). As a priori power analyses for linear mixed-effects models are not straightforward, we used a repeated-measures ANOVA with one group and three within-participant measurements as an approximation of the experimental design. Assuming a medium effect size (Cohen’s f = 0.25), an alpha level of.05, and a desired power of 90%, the analysis indicated that a minimum of 36 participants was required. This approach provides a conservative estimate of the required sample size for the planned analyses.

### Stimuli and procedure

The experiment was programmed in Vizard 7 (WorldViz, 2024) and stimuli were presented via a head-mounted-display (HTC Vive Pro Eye). We implemented two tasks: a perceptual judgment task, where only perceptual judgments were required from participants, and a pointing task, where participants were asked to perform reaching movements to anticipate where and when a given stimulus would appear. The two tasks were blocked and presented in counterbalanced order across participants.

**Fig. 1:**
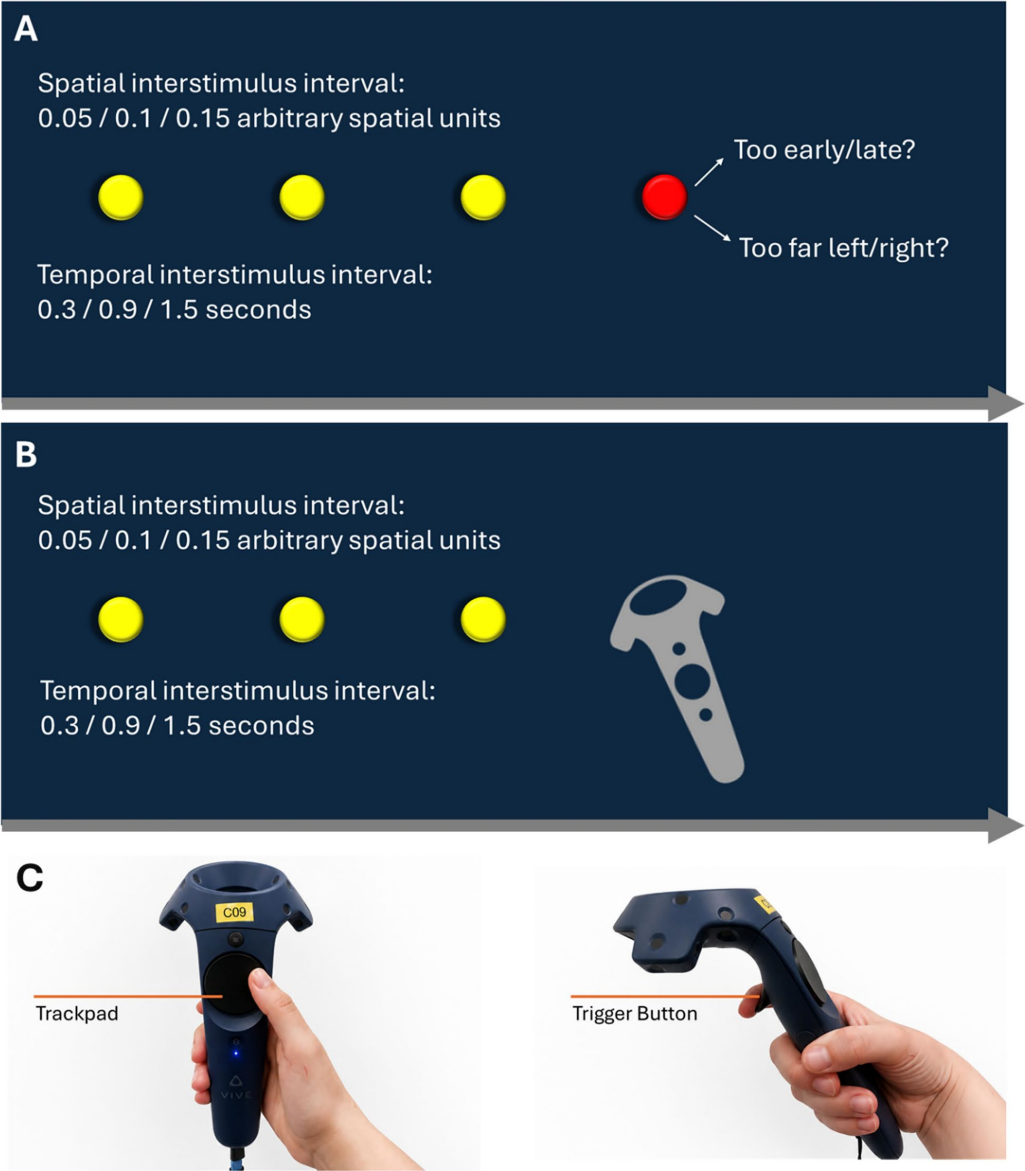
**A** Perceptual judgment task. Participants viewed a sequence of four spheres appearing consecutively. The first three spheres were yellow, and the fourth was red. After each sequence, participants judged whether the final (red) sphere appeared too early or too late in time, or too far to the left or right in space relative to the preceding three spheres.** B** Pointing task. Participants viewed a sequence of three consecutively appearing spheres. They then performed a ballistic reaching movement to indicate the time and location at which an imaginary fourth sphere would have appeared. Once they believed they had reached the correct spatiotemporal position, participants pressed the trigger button on the HTC Vive Controller, represented by the gray controller icon to the right of the spheres. **C** HTC Vive controller used in the experiment. Participants operated the trackpad with their right thumb and pressed the trigger button with their right index finger

In the *perceptual judgment task* (see Fig. 1A), participants were presented with four consecutive 3D spheres (each presented for 1 s) along an imaginary horizontal line, appearing from left to right. Temporal and spatial interstimulus intervals were constant within each trial. Across trials, temporal interstimulus intervals were either 0.3 (short), 0.9 (medium) or 1.5 (long) seconds, and spatial interstimulus intervals were either 0.05 (short), 0.10 (medium), or 0.15 (long) arbitrary virtual length units (intended to approximate 5, 10, and 15 cm respectively). This sums up to a total of 9 conditions. Participants were instructed to complete a two-alternative forced-choice task after the presentation of the fourth sphere, which was indicated by the color red (Fig. 1A). They were asked to decide whether the fourth (red) sphere had occurred too early or too late (for temporal judgments), or too far left or too far right (for spatial judgments). Which of these judgments they should make was indicated by a screen that appeared immediately after the fourth sphere, displaying either “spatial distance” or “temporal distance.” Judgments were given via the Vive controller^1^ trackpad, which is a round button with a diameter of ~ 4 cm on the top side of the controller, with a button press on the left indicating the response “too early/too far left” and a button press on the right side of the trackpad indicating the response “too late/too far right”. Button presses were executed via the right thumb. The perceptual judgment task consisted of 144 trials, with randomized spatial and temporal judgments (72 each, with each condition being presented 8 times in randomized order per spatial or temporal judgment).

The *pointing task* (Fig. 1B) looked almost identical to the perceptual judgment task, except for that participants were only presented with 3 spheres and had to infer where and when an imagined fourth sphere would appear. Participants were instructed to perform a ballistic reaching movement towards the correct location at the correct time point and press the trigger button of the Vive controller when they thought the location and timing were right. The trigger button of the Vive controller is located at the bottom side of the controller and was operated with the right index finger. Once the trigger button was pressed, both the time of the response and the controller’s position were logged. The pointing task consisted of 144 trials, with each condition being presented 16 times in randomized order.

Overall, the experiment lasted for approximately 1.5 h.

### Statistical analysis

For the perceptual judgment task, performance was measured by the number of “too late” and “too far right” responses. Trials with reaction times above 5 s were excluded from analysis (1, 2% of all trials). While initially planning on calculating two linear mixed-effects models with only a linear term for temporal/spatial stimulus interval, we opted for two generalized linear mixed-effect models where temporal/spatial stimulus interval was entered as both a linear and a quadratic term upon data inspection and model testing (see Section “Results”). The models were calculated separately for spatial and temporal judgments (R package lme4 1.1–27.1; Bates et al., 2015). For the space-on-time interference, we calculated a generalized linear mixed-effects model with a binomial error distribution and a logit link with the *temporal judgment* as the binary dependent variable, the *spatial interstimulus interval* as fixed effect, entered as both a linear and a quadratic term, and *subjects* and *temporal interstimulus interval* as random effects. The model looks as follows:

$$\small temporal\;judgment\,\sim \,spatial \, stimulus \, interval\, + \,spatial \, stimulus \, interval^{2} \, + \,\left( {1|subject} \right)\, + \,(1|temporal\;stimulus \, interval)$$

For the time-on-space interference, we calculated a generalized linear mixed-effects model with a binomial error distribution and a logit link with the *spatial judgment* as the dependent variable, the *temporal interstimulus interval* as fixed effect, entered as both a linear and a quadratic term and *subject* and *spatial interstimulus interval* as random effects. The model looks as follows:

$$\small spatial\;judgment\,\sim \,temporal \, stimulus \, interval\, + \,temporal \, stimulus \, interval^{2} \, + \,\left( {1|subject} \right)\, + \,(1|spatial\;stimulus \, interval)$$

For the pointing task, temporal performance was defined as the ratio of the theoretically correct to the observed reaction time (relative to the offset of the third sphere). Spatial performance was defined as the ratio of the theoretically correct to the observed x-coordinate between the third sphere and response location. Values above 1 indicate a bias to delayed/rightward responses and values below 1 indicate a bias to premature/leftward responses. Outliers were removed using the median absolute deviation with a threshold of 5 as implemented in R package *Routliers* (Leys et al., 2013). A total of 5.3% of data were removed on this basis. For the time-on-space interference, we calculated a linear mixed-effects model with the *spatial ratio* as the dependent variable, the *temporal interstimulus interval* as fixed effect, and *subjects* and *spatial interstimulus interval* as random effects. The model looks as follows:

$$\small spatial\;judgment\,\sim \,temporal \, stimulus \, interval\, + \,\left( {1|subject} \right)\, + \,(1|spatial\;stimulus \, interval)$$

For the space-on-time interference, we calculated a linear mixed-effects model with the *temporal ratio* as the dependent variable, the *spatial interstimulus interval* as fixed effect and *subject* and *temporal interstimulus interval* as random effects. The model looks as follows:

$$\small temporal\;judgment\,\sim \,spatial \, stimulus \, interval\, + \,\left( {1|subject} \right)\, + \,(1|temporal\;stimulus \, interval)$$

Model assumptions were visually assessed using simulation-based residual diagnostics in the *DHARMa* package (Hartig, 2016). For the perceptual judgment task models, no evidence for overdispersion or other violations of model assumptions was detected. Regarding the pointing task models, some deviation from homoscedasticity and normality were observed. However, linear mixed-effect models are robust to moderate violations of these, particularly with respect to fixed-effect estimates (Schielzeth et al., 2020).

To assess whether space–time interference in the perceptual judgment and pointing task may be based on the same underlying mechanism, we performed correlation analyses between individual interference effects in perceptual and action-related judgments. For this correlation analysis, individual linear beta parameters were extracted.

## Results

**Fig. 2:**
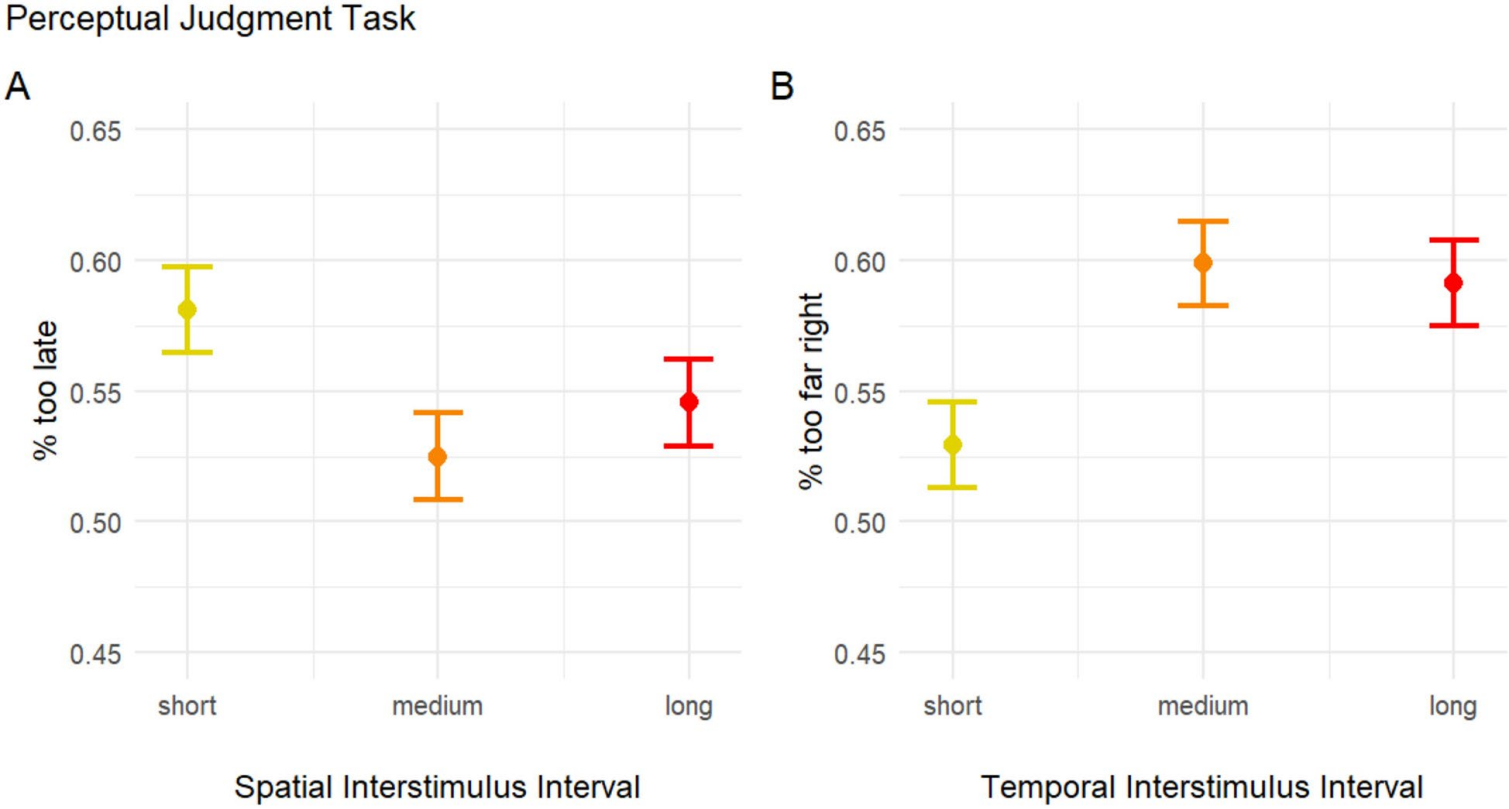
**A** Frequency of response “too late” by spatial distance. No overall bias and no linear effect of spatial distance on temporal judgments was observed, but a quadratic relationship emerged. **B** Frequency of response “too far right” by temporal distance. Overall, participants experienced the fourth sphere as too far right, and longer temporal intervals led to a relative increase of this rightward bias. Error bars indicate the mean ± the standard error

The results for the perceptual judgment task are depicted in Fig. 2 and Table 1. Overall, one-sample, two-tailed t-tests against chance level (μ = 0.5) revealed that participants tended to judge the fourth sphere as occurring too far to the right (*t*_39_ = 3.14, *p* = 0.003), but not as occurring too late (*t*_39_ = 1.70, *p* = 0.09). Although prior research on space–time interference motivated the expectation of a linear relationship between temporal and spatial intervals and judgments, visual inspection of the data (see Fig. 2) suggested a quadratic pattern. Hence, we fitted both a linear and a quadratic lmer, of which the quadratic model resulted in the better fit for both the space-on-time effect (χ^2^_1_ = 4.08, *p* = 0.043) and the time-on-space effect (χ^2^_1_ = 4.60, *p* = 0.032). Concerning the space-on-time effect (Fig. 2A), no linear relationship between spatial interstimulus interval and frequency of “too late” responses was observed, whereas the quadratic term reached significance. Regarding spatial judgments, we observed a significant linear as well as a quadratic relationship between temporal interstimulus interval and spatial judgments (Fig. 2B).

**Table 1:** Linear mixed-effects model results for the perceptual judgment task

Effect	Predictor	β	SE	z	*p*
Space-on-time (linear)	Spatial interstimulus interval	− 0.08	0.05	− 1.54	0.12
Space-on-time (quadratic)	Spatial interstimulus interval^2^	0.18	0.09	2.03	0.043
Time-on-space (linear)	Temporal interstimulus interval	0.14	0.05	2.76	0.006
Time-on-space (quadratic)	Temporal interstimulus interval^2^	− 0.19	0.09	− 2.14	0.032

**Fig. 3:**
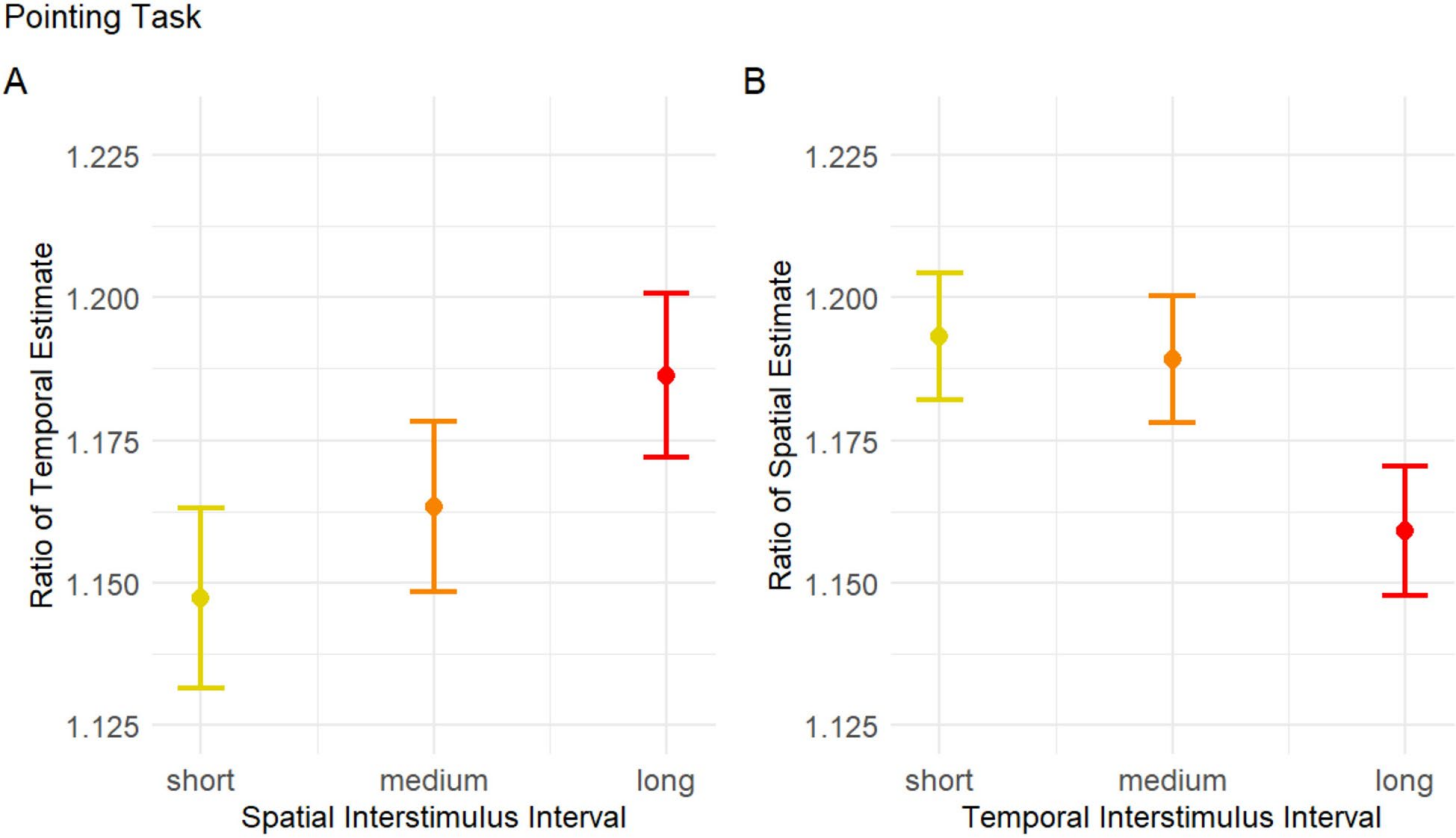
**A** Ratio of temporal responses plotted by spatial distance. Overall, participants responded too late (ratio > 1) and larger distances led to relatively later responses. **B** Ratio of spatial responses plotted by temporal distance. Although participants showed an overall rightward bias (ratio > 1), longer temporal intervals led to a relative attenuation of this rightward bias. Error bars indicate the mean ± the standard error

The results for the pointing task are depicted in Fig. 3 and Table 2. Overall, one-sample, two-tailed t-tests against theoretically perfect performance (μ = 1) revealed that participants anticipated the imagined fourth sphere too late (*t*_39_ = 3.55, *p* = 0.001) and too far to the right (*t*_39_ = 4.98, *p* < 0.001). In contrast to the perceptual judgment task, adding a quadratic term did not improve model fit for either the time-on-space effect (χ^2^1 = 0.09, *p* = 0.587) nor the space-on-time effect (χ^2^1 = 0.30, *p* = 0.321), therefore, we retained a linear model. Regarding the space-on-time effect (see Fig. 3A), we found a positive relationship between the spatial interstimulus interval and temporal prediction, indicating that participants reached for the fourth sphere relatively later in trials with larger spatial interstimulus intervals. In contrast, a negative relationship was found between the temporal interstimulus interval and prediction of the spatial location (see Fig. 3B), indicating that participants reached for the fourth sphere relatively more leftward in trials with larger temporal interstimulus intervals.

**Table 2:** Linear mixed-effects model results for the pointing task

Effect	Predictor	β	SE	t	*p*
Space-on-time (linear)	Spatial interstimulus interval	0.01	0.007	1.97	0.048
Time-on-space (linear)	Temporal interstimulus interval	− 0.02	0.006	− 2.88	0.005

Individual space–time interference effects were not significantly correlated between perceptual and action-related judgments, neither with respect to the space-on-time effect (*t*_38_ = − 0.38, *p* = 0.70,* r* = − 0.06), nor for the time-on-space effect (*t*_38_ = − 0.08, *p* = 0.94, *r* = − 0.01).

## Discussion

Space–time interference has been investigated primarily for the domain of pure perception, and studies involving goal-directed motor actions are scarce and restricted to the use of two-dimensional stimuli. Perceptual biases, however, do not necessarily generalize to motor behavior (Aglioti et al., 1995), especially when the motor behavior is done in a three-dimensional space (Freud & Ganel, 2015; Ganel et al., 2020). To address this gap, we adapted a paradigm from Schroeger et al. (2022) to examine whether space–time interference also shapes motor actions towards 3D stimuli within an immersive virtual environment. Our findings demonstrate that participants exhibited bidirectional space–time interference when performing a reaching movement to infer the location and timing of a fourth sphere following the sequential presentation of three spatially and temporally equidistant spheres.

In the pointing task, participants tended to reach for the imagined sphere overall too late, and too far to the right. Larger spatial interstimulus intervals led to an additional delay in temporal anticipation when participants reached toward an imagined fourth sphere, demonstrating a positive influence of spatial interstimulus interval on the expected timing of a fourth sphere. Conversely, larger temporal interstimulus intervals resulted in an attenuation of the rightward bias, demonstrating a negative effect of temporal stimulus interval on the expected spatial location of a fourth sphere. One plausible explanation for this negative time-on-space effect is that participants construed the sequential presentation of spheres as motion (Goldreich, 2007; Kirsch, 2023). This assumption aligns with accounts of the *tau* effect, whereby judgments of spatial distance are influenced by temporal properties (Helson, 1930; Huang & Jones, 1982). Specifically, when three stimuli are presented with constant spatial interstimulus intervals but varying temporal interstimulus intervals, relatively longer temporal intervals lead participants to judge the corresponding spatial intervals as relatively larger. The occurrence of the *tau* effect has previously been linked to the assumption of constant velocity, with the suggestion that when a constant velocity is assumed, relatively greater temporal interstimulus intervals would imply a larger traveled distance, thereby causing the illusion of a larger spatial distance (Reali et al., 2019). If participants in our experiment assumed a constant velocity of the appearing spheres, longer temporal intervals would imply slower motion within a given trial and thus lead to a leftward shift of the anticipated location, as less spatial displacement would be expected. In line with this interpretation, Riemer et al. (2026) provided evidence for the idea that the often observed asymmetric interference pattern between space and time is caused by the factor of speed (which is positively correlated with spatial distance, but negatively with temporal duration).

In addition, the negative time-on-space effect can be understood in terms of representational momentum (Hubbard, 2005, 2015), a mislocation of the final position of a moving stimulus in the direction of motion. Participants consistently overestimated spatial intervals between the third sphere and the imagined fourth sphere. The representational momentum effect decreases at lower speeds (Merz et al., 2023) and as longer temporal intervals between sphere presentations imply slower motion, this might have led to a decrease in the magnitude of the representational momentum effect. As such, the negative effect of time on space observed in our pointing task does not reflect a general leftward bias (i.e., against the direction of motion), but a relative reduction of a representational bias in the direction of motion. The observed pattern of results is consistent with these speed-related explanations. In this regard, our results converge with those of Schroeger et al. (2022) who also observed a leftward bias of spatial predictions at longer temporal interstimulus intervals. Importantly, all stimulus sequences in our experiment were presented from left to right. Research on spatial–temporal associations and cross-dimensional associations in general, as well as representational momentum accounts, suggest that intercultural differences in writing-reading direction is influential for the direction of representational biases (Dehaene et al., 1993; Fuhrman & Boroditsky, 2010; Halpern & Kelly, 1993; Santiago et al., 2007; Vallesi et al., 2014; Zebian, 2005) with stronger effects typically observed when stimuli are congruent with the learned writing-reading direction. This potentially facilitating factor needs to be considered when interpreting the results presented here, and future studies are needed to examine whether the present effects generalize to right-to-left sequences or differ across populations accustomed to different writing-reading directions.

Regarding the perceptual judgment task, participants overall judged the fourth sphere as appearing too far to the right, but not as too early or too late. Concerning the effects of time and space on each other, we observed a significant linear time-on-space effect: larger temporal interstimulus intervals increased the frequency of responding “too far right”. This observation conflicts with a finding from Merz et al. (2023) who reported a reduction of the representational momentum effect for lower implied speeds (as it was found for the pointing task in the present study). This inconsistency might be due to a lower resolution due to the dichotomic nature of perceptual judgment responses (i.e., participants could only answer “too far left” or “too far right”, whereas in the pointing task responses were assessed on a continuous scale). Additionally, the presence of a significant quadratic component suggests that the time-on-space effect varied non-linearly across temporal magnitudes. Specifically, the time-on-space effect might have been stronger for short and medium interstimulus intervals and possibly attenuated at longer intervals. In contrast, the corresponding linear space-on-time effect did not reach statistical significance. Similar to the time-on-space effect, a significant quadratic relationship suggests that spatial influence on temporal judgments may be attenuated at larger magnitudes rather than following a linear pattern. Perceptual space-on-time effects have often been reported, one prominent example being the *kappa* effect (Huang & Jones, 1982; Price-Williams, 1954). The *kappa* effect (analogous to the above-mentioned *tau* effect) describes the observation that, when three stimuli are presented sequentially with fixed temporal but varying spatial interstimulus intervals, temporal intervals accompanied by larger spatial intervals are judged as temporally longer. As the *kappa* effect has frequently been reported (De-Pra et al., 2023; Huang & Jones, 1982; Price-Williams, 1954; Reali et al., 2019), we expected spatial interstimulus intervals to significantly and linearly influence the frequency of “too late” choices. One possible explanation for the absence of such an effect is that unlike in classical studies on the *tau* and *kappa* effects, our manipulations occurred across trials rather than within a single sequence (in accordance with Schroeger et al., 2022). Hence, participants viewed spheres presented at equal spatial and temporal intervals and were then asked to judge whether the last sphere appeared too early or too late, even though the sphere always appeared at the correct (i.e., equidistant) time and location. The altered study design may have weakened a potential perceptual *kappa* effect. As noted with regard to the pointing task, the consistent left-to-right presentation may have also facilitated the observed biases in the perceptual judgment task. Future studies should test the generalizability of these effects in right-to-left contexts and across different cultural groups.

An unexpected finding was the emergence of a quadratic component in the relation between irrelevant and relevant dimensions that was found for the perceptual judgment task. This quadratic relation could indicate a reduction of space–time interference when the irrelevant temporal and spatial intervals are larger. One possible explanation is that for larger temporal and spatial magnitudes, the inherent variability in encoding these intervals (i.e., representational noise) increases, which might reduce the influence of the irrelevant dimension on judgments of the relevant dimension (Cai & Wang, 2022; Riemer & Cai, 2024).

Although the space-on-time effect did not reach a significant level, the descriptive direction of the linear space-on-time as well as time-on-space effects in the perceptual judgment task mirror the direction of effects in the pointing task: For the space-on-time effect, short spatial interstimulus intervals were associated with a descriptive increase of “too late” judgments compared to medium and large spatial interstimulus intervals, implying that participants expected the fourth sphere to appear earlier in time when spatial interstimulus interval was smaller. When reaching for the fourth imagined sphere in the pointing task, participants showed the complementing pattern. Shorter spatial interstimulus intervals were associated with participants reaching for the sphere earlier in time compared to longer spatial intervals. As for the time-on-space effect, in the perceptual judgment task greater temporal interstimulus intervals led to participants judging the location of the fourth sphere as “too far right” more frequently, implying that participants expected the sphere more leftward with increasing temporal interstimulus intervals. Accordingly, in the pointing task, participants demonstrated a spatial shift to the left of the anticipated location when the temporal interstimulus interval increased. Although the results from the perceptual judgment tasks complemented those from the pointing task, we did not observe a correlation between the two tasks, leaving open the question of whether perceptual and motor-related space–time interference is based on a common mechanism.

It has been suggested that motor timing relies on different mechanisms from those underlying mere perceptual timing (Bueti et al., 2008; De Kock et al., 2021). This idea resonates with the proposal that the processing of stimulus features differs depending on whether the information is used for perception and action (Goodale & Milner, 1992; Milner & Goodale, 2008). Also beyond the domains of time perception, converging evidence suggests the existence of distinct neuronal pathways for the processing of action-related versus action-unrelated information (Bublatzky & Riemer, 2025; Dijkerman & de Haan, 2007; Ganel et al., 2008; Goodale et al., 1991; Kammers et al., 2006). In light of these differences between neuronal processing for action and perception, the finding that space–time interference (reported in many perceptual paradigms) extends to the execution of reaching movements towards 3D stimuli is of particular relevance. Our everyday life frequently requires the integration of temporal and spatial information to guide motor behavior, such as reaching for objects, catching objects, or avoiding obstacles while walking. Perceptual space–time interference has been demonstrated in all stages of life, from newborns (de Hevia et al., 2014) to childhood (Casasanto et al., 2010; Hallez & Balcı, 2024; Srinivasan & Carey, 2010) to young adulthood and advanced age (Bogon et al., 2025; Cai et al., 2018; Jagorska et al., 2025; Riemer et al., 2022). Moreover, previous work suggests that perceptual space–time interference may be increased in early childhood (Hallez & Balcı, 2024) and increase again with advanced age (Jagorska et al., 2025), highlighting the importance of investigating whether similar increases emerge in motor behavior. Especially for older adults, increased space–time interference in motor behavior might provoke difficulties in timing their steps precisely while crossing a busy street or avoid obstacles while walking, which can increase the risk of falls.

Importantly, with regard to the temporal domain, the current study employed an explicit timing paradigm in which the participants’ attention was explicitly directed to the implemented temporal intervals. In contrast, most real-world movements rely on implicit timing, where temporal information is processed automatically without focused attention (Capizzi et al., 2022; Herbst et al., 2022; Zelaznik et al., 2002). Thus, while our results demonstrate that space–time interference occurs in motor behavior, further research using more naturalistic, implicit timing paradigms is necessary to determine how these effects generalize to everyday behavior. Additionally, it should be noted that our paradigm did not allow for a strict dissociation of perceptual and motor processes. The perceptual judgment task, while emphasizing perceptual judgments, nevertheless required a motor response in the form of a click on the Vive Controller trackpad. Conversely, the pointing task inherently relied on perceptual processing in addition to motor execution. Therefore, the present study does not constitute a pure perception-versus-action comparison. Rather, both tasks involved a combination of perceptual and motor components, with each task emphasizing different aspects more strongly.

## Conclusion

In the present study we demonstrate that space–time interference occurs not only when making perceptual judgments, but also when performing goal-oriented reaching movements towards 3D objects presented within immersive virtual reality. This finding illustrates that everyday behaviors such as catching a ball or navigating traffic might be susceptible to cross-dimensional interference between temporal and spatial processing. As no correlation between the biases in perceptual and motor judgments was found, the question about a common neuronal mechanism for the emergence of perceptual and motor-related space–time interference remains open.

## Data Availability

The datasets analyzed during the current study are available in the GitHub repository, https://github.com/cindyjag/SpaceTimeInterferenceInAction.

## Footnotes

1 https://www.vive.com/eu/accessory/controller/
